# From contiguity to accuracy: Validation-centered perspectives on bacterial genome assembly

**DOI:** 10.71150/jm.2604004

**Published:** 2026-06-19

**Authors:** Minkyung Kim, Yong-Joon Cho, Ok-Sun Kim

**Affiliations:** 1 Division of Life Sciences, Korea Polar Research Institute, Incheon 21990, Republic of Korea; 2 Department of Molecular Bioscience, Kangwon National University, Chuncheon 24341, Republic of Korea; 3 Multidimensional Genomics Research Center, Kangwon National University, Chuncheon 24341, Republic of Korea

**Keywords:** genome sequencing, genome assembly, assembly validation, assembly algorithms, genome accuracy, draft genome

## Abstract

Recent advances in sequencing technologies, particularly long-read platforms, have substantially improved contiguity of bacterial genome assemblies and enabled the routine generation of near-complete or circular genomes. However, achieving a contiguous assembly does not necessarily guarantee accuracy. Assembly errors, including structural misassemblies, collapsed repeats, incorrect circularization, plasmid reconstruction errors, and nucleotide-level inaccuracies, remain prevalent and may lead to misleading biological interpretations if not properly identified. In this review, we provide a comprehensive overview of bacterial genome assembly from a validation-centered perspective and examine the underlying causes of draft genome formation and assembly uncertainty, highlighting the roles of repetitive genomic structures, platform-specific error profiles, and algorithmic limitations. We further emphasize that the central challenge in contemporary bacterial genomics is no longer simply to maximize assembly contiguity, but to determine whether apparently complete genomes are truly correct and sufficiently reliable for their intended downstream applications. We propose a practical decision-making framework that links sequencing strategy, assembly workflow, polishing, and validation rigor, and introduce a tiered confidence classification to guide the interpretation of genome assembly reliability. As bacterial genome sequencing becomes increasingly routine and large-scale, future efforts should prioritize accuracy, reproducibility, transparent reporting, and evidence-supported validation over completeness alone.

## Introduction

The initiation of large-scale genome sequencing projects in the early 1990s led to a rapid expansion of microbial genome sequencing efforts, thereby contributing substantially to the advancement of environmental microbiology (Bentley et al., 2003; Eiglmeier et al., 2001; Glaser et al., 1993). The introduction of next-generation sequencing (NGS) platforms, particularly Illumina, enabled the generation of large volumes of highly accurate short-read data (Metzker, 2010). However, the limited read length of these platforms has posed challenges in resolving repetitive and structurally complex genomic regions (Koren et al., 2013; Rhoads and Au, 2015). The development of third-generation sequencing technologies in the 2010s, such as Pacific Biosciences (PacBio) and Oxford Nanopore Technologies (ONT), has significantly improved genome assembly contiguity by producing long reads capable of spanning repetitive regions and structural variations (Koren et al., 2013; Reuter et al., 2015). Consequently, the number of complete bacterial genomes reported in public databases has increased substantially, with over 77,000 complete genomes currently available.

Despite these advances, an important question remains: can all reported complete circular genomes be considered accurate representations of the true genome structure? Genome assembly is a complex computational process that reconstructs chromosome-scale sequences from fragmented reads. In the absence of a suitable reference genome, de novo assembly is typically employed, and its accuracy is influenced by multiple factors, including sequencing technology, read length, sequencing depth, and assembly algorithms. With the rapid development of sequencing technologies, a wide range of genome assembly algorithms and software tools have been introduced; however, different assemblers can produce varying results even when applied to the same dataset (Johnson et al., 2023; Rojas-Miranda et al., 2025; Trisakul et al., 2024). These discrepancies may lead to differences in genome structure, contiguity, and the presence of assembly artifacts, raising concerns about the reliability of assembled genomes. Such uncertainties are particularly critical when genome assemblies are used for downstream analyses, including comparative genomics, functional annotation, and evolutionary studies.

To address these challenges, evaluation frameworks based on contiguity, completeness, and correctness (the 3C criteria) have been proposed to systematically assess assembly quality (Molina-Mora et al., 2020). Contiguity describes the degree of assembly fragmentation and is commonly assessed using metrics based on contig number and size, including maximum contig length, total assembly length, and N50. Completeness can be defined along two complementary dimensions: structural completeness, which refers to the reconstruction of an entire chromosome into a single circular contig, and gene content completeness, typically evaluated using marker gene-based approaches such as BUSCO or CheckM. Notably, these dimensions do not necessarily coincide. Accuracy, often discussed alongside correctness in assembly evaluation frameworks, encompasses both structural accuracy, reflecting the absence of misassemblies such as inversions or rearrangements, and base-level accuracy, defined by the absence of nucleotide substitutions and indels. These approaches aim not only to achieve structural completeness but also to ensure a “truly complete genome” satisfying all 3C criteria, whereby assembled genomes accurately reflect the underlying biological sequences.

Existing reviews have largely focused on sequencing platforms, general assembly algorithms, or broad benchmarking of assembly tools. In contrast, this review specifically addresses bacterial genome assembly from the perspective of validation, emphasizing the gap between apparent completeness and actual correctness. Given that long-read sequencing, which improves assembly continuity, has become a primary approach for genome assembly, this review places greater emphasis on long-read and hybrid assembly strategies. We discuss how repetitive genomic structures, sequencing biases, and assembler-specific behaviors contribute to draft genome formation and assembly errors, and we highlight why these issues remain important even in highly contiguous long-read assemblies. We further argue that, as bacterial genome sequencing rapidly expands and assembled genomes are increasingly used in comparative, functional, and surveillance studies, rigorous validation is becoming more urgent rather than less. By integrating current knowledge on assembly challenges, tool variability, and validation strategies, this review provides a framework for evaluating the reliability of bacterial genome assemblies beyond contiguity alone.

## Structural Ambiguity Arising from Complex Genome Architecture

**Fig. 1.**
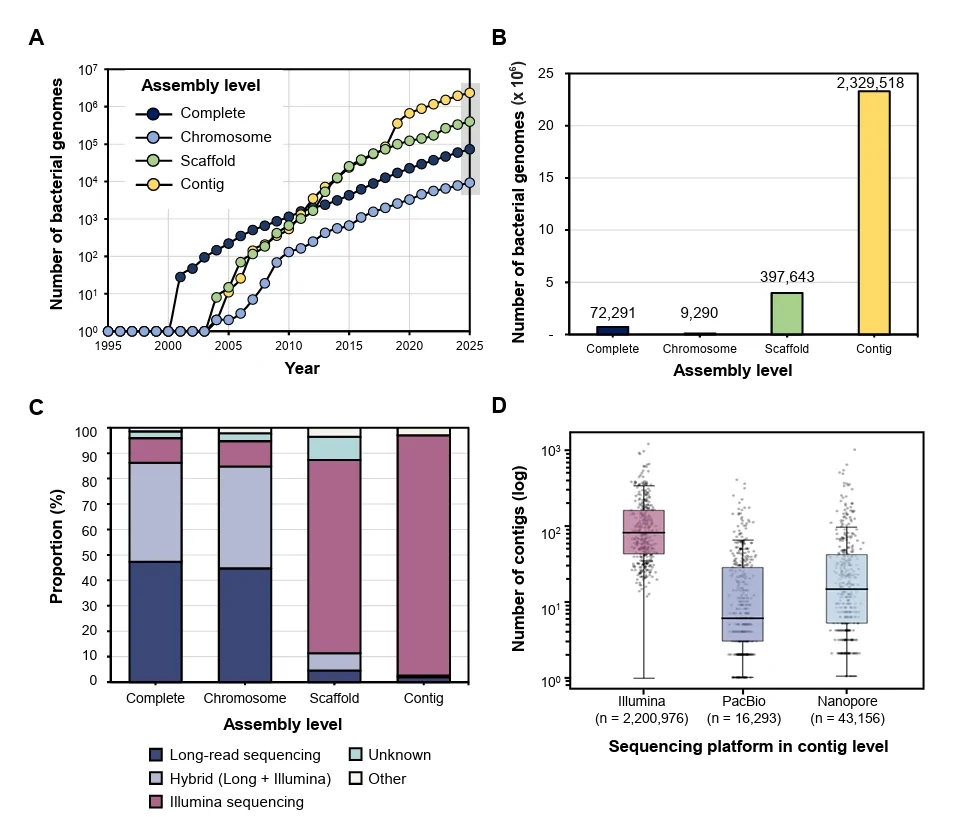
Overview of bacterial genome assemblies and sequencing platforms. (A) Number of bacterial genomes by assembly level deposited in NCBI from 1995 to 2025, with the y-axis shown on a logarithmic scale. (B) Distribution of bacterial genomes by assembly level in 2025. (C) Proportion of sequencing platforms (Illumina, PacBio, and ONT) used for bacterial genome assemblies across different assembly levels. (D) Distribution of contig counts according to sequencing platform for genomes at the contig assembly level.

Advances in high-throughput sequencing technologies have led to a rapid expansion of genomic data, with approximately 3 million bacterial genomes currently available in public databases (NCBI, 2026) (Fig. 1A and 1B). These advancements have improved read length, accuracy, and throughput, thereby enabling the generation of increasingly contiguous genome assemblies (Loman and Pallen, 2015). However, as of 2025, the number of contig-level assemblies exceeds that of complete genomes by approximately 32-fold (Fig. 1B). Long-read sequencing generally reduces contig fragmentation; however, draft assemblies remain common even when long-read data are used (Fig. 1C and 1D). Recent studies have shown that assembly breakpoints frequently occur at repetitive regions, and that some of these repeats, particularly long and highly similar sequences, can lead to misassemblies even in genomes previously reported as complete (Acuña-Amador et al., 2018).

Repetitive sequences vary widely in length, ranging from short dinucleotide repeats to long segments spanning several kilobases (Treangen et al., 2009). Historically, genomes containing repeat regions longer than the typical size of rRNA operons (~5–7 kb) were difficult to assemble into complete genomes using earlier sequencing technologies (Koren et al., 2013). With the advancement of third-generation sequencing platforms, increasingly long repeat structures have been reported, including inverted repeats exceeding tens of kilobases, such as those identified in *Lactobacillus* species (Colombini et al., 2025; El Kafsi et al., 2017). These findings highlight that assembly difficulty is not uniform across species but is strongly influenced by genome architecture.

**Fig. 2.**
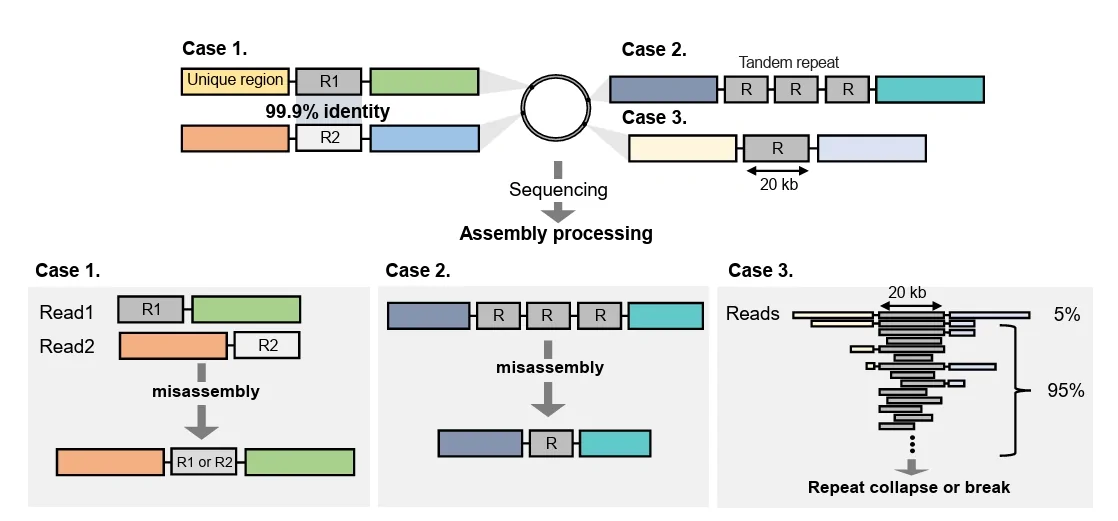
Mechanism of repeat-induced assembly ambiguity and misassembly in long-read sequencing. Case 1. Highly identical repeats. Two repeat copies (R1 and R2) with near-identical sequences (~99.9% identity) are flanked by distinct genomic regions. Although reads may partially span these regions, the lack of sufficient sequence divergence prevents unique assignment of reads to specific repeat copies, resulting in ambiguous assembly paths and potential misassembly. Case 2. Tandem repeat structures. Multiple consecutive repeat units arranged in tandem can lead to uncertainty in repeat copy number during assembly. Depending on the algorithm and supporting evidence, repeat arrays may be collapsed into fewer copies, producing assemblies that underestimate the true genomic length. Case 3. Read length distribution effects. Even when ultra-long reads are present, only a fraction of reads may exceed the length of long repeat regions (e.g., 20 kb). When the majority of reads are shorter than the repeat, insufficient spanning evidence can lead to repeat collapse or fragmentation at repeat boundaries.

Although long reads can span repetitive regions, when multiple repeat copies share nearly identical sequences (e.g., > 99–99.9% identity), reads traversing these regions may lack sufficient sequence divergence to uniquely assign their genomic origin (Fig. 2, case 1). Such regions therefore collapse into a single node in the assembly graph, generating ambiguous connections between distinct genomic contexts and leading to incorrect path reconstruction. Tandem repeats comprising two or more nearly identical copies are frequently reduced to a single representation during assembly (Fig. 2, case 2). As a consequence, assemblies can appear circular and complete yet be shorter than the true genome, containing hidden structural errors that are not detectable using standard contiguity metrics alone. When repetitive regions exceed the effective read length or lack sufficient unique flanking sequences, assembly algorithms often fail to resolve their correct genomic placement, resulting in fragmentation or structural misassemblies (Fig. 2, case 3; Waters et al., 2025). Genomes containing large prophage insertions, genomic islands, or multiple highly similar rRNA operons therefore remain challenging to resolve with respect to 3C criteria, particularly in terms of structural correctness.

Plasmids represent inherently dynamic genetic elements that vary in copy number, size, and distribution across cells, often contributing to heteroresistance and population-level genomic heterogeneity (Shintani et al., 2015). These biological characteristics can lead to uneven representation of plasmid sequences and introduce ambiguity in assembly graphs. Insertion sequences (IS elements), transposons, and antimicrobial resistance cassettes are frequently shared between chromosomal and plasmid contexts, such that reads derived from these regions cannot be uniquely assigned to a specific replicon (Carattoli et al., 2014). Moreover, homologous IS elements and transposons shared among plasmids can give rise to chimeric plasmid contigs or misassemblies, resulting in underestimation of plasmid number or structural complexity. In addition to sequence homology, variation in plasmid copy number presents a further challenge. Low-copy or single-copy plasmids often display coverage similar to that of the chromosome, increasing the likelihood of being obscured by background signal or fragmented due to insufficient read support (Antipov et al., 2016a). This challenge is further exacerbated when multiple plasmids with different copy numbers coexist within the same cell, complicating accurate resolution and separation.

These limitations collectively give rise to the so-called “false completeness” problem, whereby assemblies appear structurally complete despite containing unresolved errors that are not captured by contiguity-based metrics. Such ambiguities can directly affect genome size estimation and lead to inconsistencies among assemblies generated using different computational approaches.

## Limitations of Long-Read Sequencing Accuracy

PacBio high-fidelity (HiFi) and ONT long-read sequencing represent two dominant contemporary platforms, offering a fundamental trade-off between base-level accuracy and maximum read length. In 2019, PacBio introduced HiFi sequencing, which addressed several limitations of earlier long-read technologies and has since become a widely adopted platform for modern genome sequencing. PacBio HiFi reads are generated through circular consensus sequencing (CCS), in which multiple passes over the same DNA molecule are combined to produce highly accurate consensus sequences. As a result, read lengths are typically constrained to approximately 10–25 kb, but achieve high accuracy exceeding Q30 (> 99.5%) (Hon et al., 2020; Travers et al., 2010). In contrast, ONT sequencing analyzes native DNA molecules in a single pass and can therefore generate ultra-long reads that frequently exceed 100 kb, thereby improving structural resolution across complex genomic regions, albeit with higher residual error rates (Peng et al., 2025).

These platforms also differ in their characteristic error profiles. PacBio sequencing errors primarily arise from misinterpretation of fluorescence signals and temporal variation in polymerase kinetics. In HiFi sequencing, these errors are largely random and are substantially reduced during CCS consensus generation, resulting in error rates below 1% (Wenger et al., 2019). Nevertheless, homopolymeric regions remain a persistent challenge, as accurately determining the exact number of consecutive identical bases across multiple passes is still difficult, often leading to short insertion-deletion errors (Hu et al., 2024a). ONT sequencing, which infers nucleotide identity from ionic current signals, exhibits even greater difficulty in homopolymeric tracts than PacBio (Bouras et al., 2024b; Chiou et al., 2023). Accuracy declines markedly when homopolymers exceed five bases, and the profile of substitution errors can further vary with GC content and base modifications, including methylation (Delahaye and Nicolas, 2021).

These differences have important implications for genome assembly. The shorter but highly accurate HiFi reads are generally more effective for resolving small-scale sequence variation and reducing base-level errors, whereas the substantially longer ONT reads provide superior ability to span large repetitive regions and structural variants. Nevertheless, residual errors from both platforms can compromise repeat resolution and lead to misassemblies, particularly in regions with high sequence similarity (Phillippy et al., 2008; Treangen and Salzberg, 2011). Indel errors, even at low frequency, can introduce frameshifts or premature stop codons, thereby affecting downstream gene prediction and functional annotation. Notably, in a genome the size of *Escherichia coli* (~4.6 Mb), even a 0.5% error rate corresponds to more than 23,000 incorrect bases, highlighting the substantial absolute number of errors. Such errors can substantially compromise protein-coding gene prediction by introducing frameshifts and premature stop codons, thereby resulting in inaccurate estimates of both gene content and functional potential and highlighting the importance of systematic assembly validation and, when necessary, manual curation (Watson and Warr, 2019).

## Challenges and Limitations of Genome Assembly Algorithms

Genome assembly algorithms are fundamental to genome reconstruction; however, their inherent limitations critically constrain the accuracy and completeness of assembled genomes. Assembly algorithms play a central role in determining the structure and quality of the reconstructed genome (Espinosa et al., 2023; Merda et al., 2024; Rojas-Miranda et al., 2025; Tizabi et al., 2022). These algorithms can be broadly classified into overlap-based approaches and graph-based approaches, each optimized for different sequencing technologies and error profiles. The overlap–layout–consensus (OLC) methods reconstruct genomes by identifying pairwise overlaps between reads and generating a consensus sequence, making them well suited for long-read data (Miller et al., 2010; Myers, 2005). However, high sequencing error rates can complicate accurate overlap detection, potentially leading to misassemblies, representing a fundamental challenge in accurately reconstructing genome structure. In contrast, de Bruijn graph (DBG)-based methods decompose sequencing reads into shorter k-mers and construct graphs based on exact sequence matches, enabling efficient assembly of short-read data (Bankevich et al., 2012; Simpson et al., 2009; Zerbino and Birney, 2008). However, the reliance on fixed k-mer sizes limits the resolution of repetitive regions and sensitivity to uneven sequencing coverage, often resulting in fragmented and ambiguous assemblies (Nagarajan and Pop, 2013). Although extensions such as string graphs, repeat graphs, and fuzzy Bruijn graphs have been developed to address these limitations, highly identical repeats and complex genomic structures remain difficult to resolve (Jain, 2023; Kolmogorov et al., 2019; Myers, 2005; Ruan and Li, 2020).

The diversity of assembly tools reflects fundamental differences in algorithmic design (Table 1). Early assemblers developed for Sanger or short-read sequencing data, including DBG-based tools such as Velvet, SOAPdenovo, and SPAdes, are optimized for handling large volumes of short, high-accuracy reads (Li et al., 2010; Peng et al., 2010; Zerbino and Birney, 2008). Long-read based assemblers adopt OLC-based strategies to leverage long reads spanning repetitive regions, thereby improving assembly contiguity (Chen et al., 2021a; Koren et al., 2017; Li, 2016; Vaser and Šikić, 2021). Long-read assembly has reduced the fragmentation typical of short-read assemblies, yet substantial inter-assembler variability persists even when identical long-read datasets are used. Small plasmids are frequently underrepresented or entirely missed in assemblies generated from long-read data. Although this limitation is partly attributable to biases introduced during library preparation (Wick et al., 2021b), the present review focuses specifically on differences among assemblers. Substantial variability has been reported for small plasmid recovery across long-read assemblers (Flye, 67–79%; Miniasm, 64%; Raven, 39%), whereas the hybrid assembler Unicycler, which integrates short-read data, achieved complete recovery (Johnson et al., 2023). Additionally, in a benchmark of eight long-read assemblers for prokaryotic genomes, Flye and Canu were generally reliable but differed in circularization behavior (Wick and Holt, 2021). Flye (and Raven) frequently produced assemblies with terminal sequence truncation, whereas Canu (and NextDenovo) often retained terminal overlaps, leading to artificial sequence duplication at circular boundaries. Also, Miniasm/Minipolish most consistently achieved clean circularisation; NextDenovo/NextPolish performed well for chromosome completion but poorly for plasmid recovery. These differences arise from fundamental distinctions in algorithmic design rather than incidental implementation details (Trisakul et al., 2024). Long-read assemblers differ in how they correct noisy reads, represent assembly graphs, resolve repeats, derive consensus sequences, and handle circular replicons (Amarasinghe et al., 2020; Wick and Holt, 2021). Because long-read data still retain substantial indel-heavy and homopolymer-associated errors, assembly quality remains strongly influenced by the interaction between read error profiles and tool-specific correction and polishing strategies. Thus, high contiguity does not necessarily reflect superior base-level accuracy, nor does chromosomal completion ensure correct circularization or complete recovery of all replicons.

To further mitigate the limitations of individual sequencing technologies, hybrid assembly approaches have been developed to integrate short and long reads within a single workflow. These approaches can be broadly categorized into algorithm-integrated assemblers and pipeline-based frameworks. Algorithm-integrated methods, such as hybridSPAdes, extend DBG-based assembly by incorporating long-read information directly into the graph structure (Antipov et al., 2016b). In contrast, pipeline-based approaches, including Unicycler, Hybracter, and WENGAN, combine multiple assemblers and post-processing steps such as scaffolding and polishing (Di Genova et al., 2021; Wick et al., 2017). Unicycler uses SPAdes as its core short-read assembler and integrates additional steps, including long-read bridging, graph simplification, and polishing, to generate complete assemblies (Wick et al., 2017). In contrast to Unicycler, Hybracter implements a long-read-first assembly framework in which long reads are initially assembled using Flye, followed by iterative polishing with long-read (Medaka) and short-read tools (Polypolish and Pypolca) (Bouras et al., 2024a). In benchmarking analyses, Hybracter hybrid produced near-zero error rates, with median counts of 0 single nucleotide variants (SNVs) and 0 small indels, compared to substantially higher error rates observed in Unicycler assemblies (median 34 SNVs and 11 indels). In addition to improvements in chromosome-level accuracy, Hybracter incorporates a dedicated plasmid assembly module, Plassembler, enabling more complete and accurate recovery of plasmid sequences. While hybrid assembly strategies substantially improve genome accuracy, the integration of multiple tools and polishing steps introduces dependencies on sequencing depth and data quality, which can influence the final assembly outcome. Furthermore, certain polishing tools may introduce errors under specific conditions, and therefore hybrid approaches do not guarantee completely error-free assemblies.

Additionally, tools implementing consensus-based approaches, such as Trycycler and Autocycler, have been developed to integrate multiple independent long-read assemblies and reduce tool-specific stochastic variation (Wick et al., 2021a, 2025). However, when assemblers share similar algorithmic assumptions or error profiles, their outputs can converge on the same incorrect structure, allowing systematic errors to persist in the final consensus. In practice, Trycycler also requires manual intervention to resolve irreconcilable graph structures, which limits scalability and introduces operator-dependent variability (Wick et al., 2021a). Therefore, consensus-based assembly should not be considered a substitute for validation, but rather as a complementary strategy that addresses inter-assembler variability without independently confirming biological accuracy.

## Polishing and Validation Approaches for Genome Assembly

As the importance of genome sequence accuracy and assembly reliability has become increasingly recognized, a variety of tools have been developed to improve genome assemblies through polishing, assess assembly quality, and resolve assembly-related errors (Tables 2 and 3). The performance of polishing tools varies substantially depending on their underlying algorithms, data integration strategies, and computational requirements. In a comparative analysis of parameterized polishing tools applied to ONT assemblies from nine bacterial genomes, Polypolish-careful alone is recommended under conditions of extremely low sequencing depth (< 5×) or when minimizing false-positive corrections is a primary concern, whereas Pypolca-careful is recommended for single-nucleotide polishing in all other scenarios (Bouras et al., 2024b). In contrast, other tools (e.g., Medaka, NextPolish, and Pilon) were reported to introduce additional errors under suboptimal conditions, particularly at low sequencing depths. Complementary studies further indicate that long-read–based polishing tools, such as Racon, Medaka, and DeepPolisher, improve consensus accuracy by leveraging long-read data; however, their performance generally remains inferior to that of hybrid polishing approaches integrating both short- and long-read datasets (Lee et al., 2021). Consistent with these observations, tests of 132 combinations of assembly and polishing tools demonstrated that polishing performance is largely determined by tool combinations and pipeline design rather than by the choice of a single tool (Luan et al., 2024). Notably, the order of polishing steps is critical, with the best-performing pipeline applying long-read polishing using Medaka followed by short-read polishing with tools such as NextPolish. These findings collectively indicate that achieving high-confidence genome assemblies requires not only careful selection and combination of tools but also independent validation to ensure that residual errors are minimized.

Deep learning-based approaches, including Medaka and DeepPolisher, leverage patterns in read alignments to improve base-level accuracy, effectively resolving sequencing errors in many contexts. However, given that polishing tools are primarily designed for base-level correction and offer limited capacity for resolving structural errors, independent validation of assembly structure is essential. Among available strategies, read mapping is one of the most widely used approaches for assessing structural consistency. By aligning raw sequencing reads back to the assembled genome, it is possible to identify discrepancies such as mismatches, coverage gaps, and abnormal coverage patterns that may indicate assembly errors (Bzikadze et al., 2022; Firtina et al., 2020; Li et al., 2023). Regions with unusually high or low coverage can reflect repeats, duplications, or missing sequences (Gao et al., 2019). However, such signals may also arise from biological features or sequencing biases, and therefore require careful interpretation (Delahaye and Nicolas, 2021; Gunasekera et al., 2021). When a closely related reference genome is available, alignment-based approaches such as QUAST and Assemblytics provide effective means of assessing structural accuracy (Gurevich et al., 2013; Nattestad and Schatz, 2016). Comparative analysis of assembled genomes against reference sequences enables the identification of large-scale structural discrepancies, including inversions, translocations, insertions, and deletions, as well as inconsistencies that are not captured by contiguity metrics alone. However, reference-based validation is susceptible to bias when the reference genome contains errors or differs substantially from the target genome (Sousa et al., 2019). Additionally, evolutionary divergence is often misinterpreted as structural variation, necessitating careful interpretation. In this context, graph-based inspection has also emerged as a valuable strategy for identifying unresolved or ambiguous regions. Tools such as Bandage and gfatools enable visualization and interrogation of assembly graphs, allowing direct observation of branching structures, bubbles, and cycles that reflect uncertainty in the assembly (Marijon et al., 2019; Wick et al., 2015). As such, graph inspection can provide critical insights into regions requiring further validation or manual curation.

In addition to these approaches, comprehensive assessment of assembly quality requires evaluation of completeness, structural consistency, and biological plausibility. Assembly completeness and contamination represent fundamental aspects of genome quality assessment. Marker gene–based approaches, such as those implemented in BUSCO and CheckM, are widely used to estimate completeness by assessing the presence of conserved single-copy genes (Parks et al., 2015; Simão et al., 2015). However, this approach relies on the assumption that conserved single-copy genes are stably maintained across lineages; violations of this assumption in genomes shaped by extensive horizontal gene transfer or lineage-specific gene expansions can result in over- or underestimation of completeness. Additionally, the predefined marker gene sets used by these tools do not uniformly represent all bacterial clades, which can introduce lineage-dependent biases in the completeness estimates. Therefore, the completeness and contamination indicators derived from these approaches should be interpreted in the context of the evolutionary background of the organism and in combination with lines of complementary evidence.

Importantly, reliance on a single validation strategy can lead to false confidence in assembly correctness, particularly in repeat-rich or structurally complex genomes. Although polishing effectively corrects small-scale nucleotide errors, it does not resolve structural inaccuracies. Similarly, mapping-based approaches often fail to detect errors in repetitive regions, and the resulting signals do not always clearly distinguish between biological variation and assembly artifacts. In particular, some assembly errors persist even after multiple rounds of validation and polishing, especially in complex genomic regions. These limitations underscore the necessity of applying multiple complementary validation strategies to achieve high-confidence genome assemblies. A minimum validation checklist should therefore include: (i) read remapping to evaluate coverage uniformity and identify potential misassemblies, (ii) cross-assembler comparison or consensus-based approaches to assess structural consistency, (iii) inspection of assembly graphs to resolve ambiguous repeat junctions, and (iv) marker gene-based evaluation of completeness and contamination. While these approaches provide a robust foundation for validation, defining universally applicable thresholds remains challenging due to variability in sequencing technologies, genome complexity, and dataset-specific characteristics. Nevertheless, practical benchmarks can serve as general guidance. For example, base-level accuracy is often considered high when quality values (QV) approach or exceed ~50, whereas marker gene-based completeness above 95% and contamination below 2% are commonly used as indicative criteria for high-quality bacterial genomes. However, such thresholds should be interpreted cautiously and in context, rather than as absolute indicators of correctness.

Building on these considerations, we propose a tiered validation framework (bronze, silver, and gold) to facilitate practical assessment of genome assembly reliability based on validation rigor rather than sequencing technology alone. Assemblies classified as bronze represent those generated using a single assembler and subjected to polishing and basic statistical evaluation (e.g., contiguity metrics and marker gene completeness), but lacking thorough validation of structural and base-level accuracy. Assemblies classified as silver include additional validation steps, such as cross-assembler comparison, read mapping-based coverage assessment, and graph-based inspection of structurally ambiguous regions, thereby providing improved confidence in structural integrity. Finally, gold-level assemblies represent the highest-confidence genomes, for which all validation steps have been performed, including independent reference comparison and/or manual curation to verify both structural correctness and base-level accuracy. Importantly, this classification framework is intended to reflect the depth and confidence of validation rather than the sequencing strategy itself.

## A Practical Framework for High-Confidence Complete Genomes

**Fig. 3.**
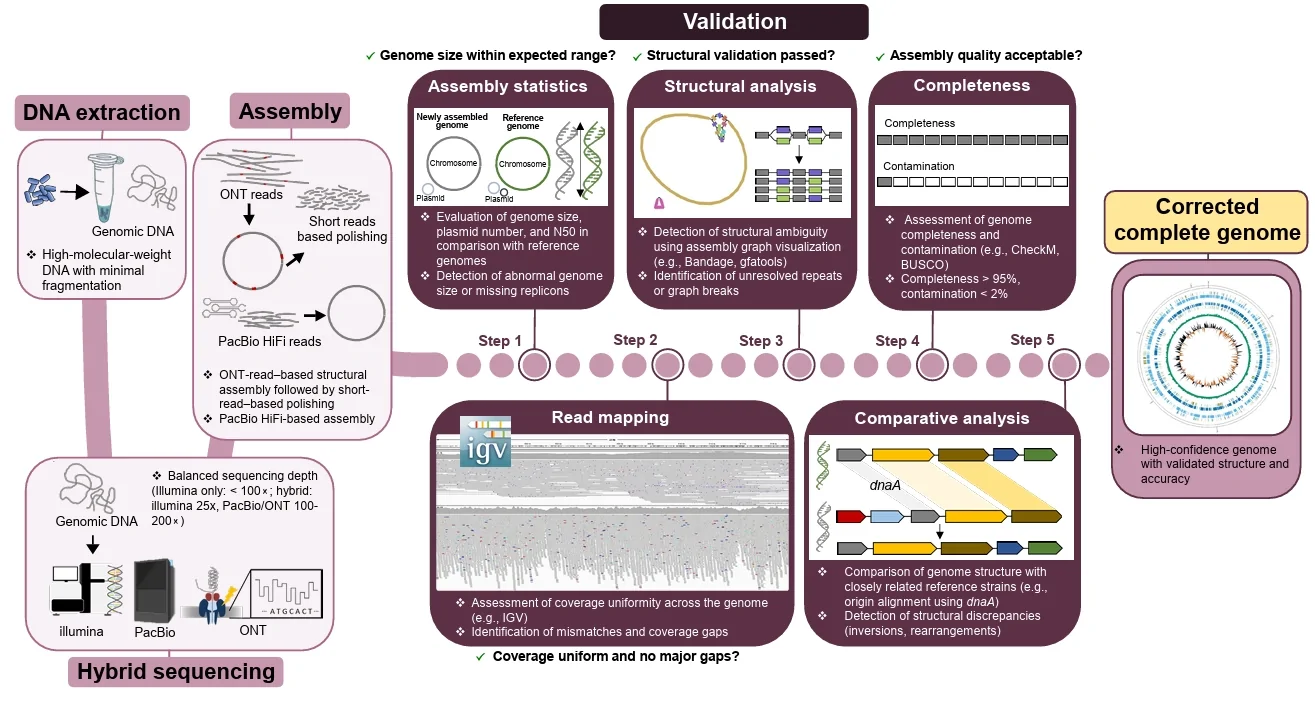
Workflow for generating a high-confidence complete bacterial genome. Schematic overview of genome assembly and validation. High-molecular-weight DNA is subjected to hybrid sequencing using short- and long-read platforms. Assembly is performed using either an ONT-based hybrid approach, in which ONT structural reconstruction is followed by short-read polishing, or a PacBio HiFi-based approach. Validation includes evaluation of assembly statistics (genome size, plasmids, N50), read mapping for coverage assessment, assembly graph inspection for structural ambiguity, completeness and contamination assessment (e.g., CheckM, BUSCO), and comparative analysis with reference genomes (e.g., *dnaA* alignment) to detect structural discrepancies such as inversions and rearrangements.

Obtaining complete genome assemblies requires not only appropriate sequencing technologies but also well-designed experimental procedures and careful selection of computational tools. We propose an integrated framework that encompasses multiple stages, from sample preparation to iterative validation, to achieve reliable genome reconstruction (Fig. 3). However, because multiple variables such as genome complexity and research objectives can influence sequencing and assembly strategies, this should be regarded not as a fixed rule but as a conditional decision-making framework.

A critical first step is the extraction of high-quality, high-molecular-weight DNA. In bacterial genome studies, the use of cultures derived from a single colony is generally recommended to minimize genomic heterogeneity (Wick et al., 2023). Fragmentation of DNA during extraction can limit the effective read length, particularly for long-read sequencing technologies, thereby reducing the ability to resolve repetitive genomic regions. To minimize DNA shearing, harsh mechanical handling such as excessive vortexing, pipetting, and repeated freeze–thaw cycles should be avoided (Branton and Deamer, 2019). In addition, when performing hybrid assembly using multiple sequencing platforms, it is preferable to use a single DNA extract to maintain consistency across datasets (Wick et al., 2023). Therefore, the use of intact, high-quality DNA is essential for achieving highly contiguous assemblies.

Sequencing design and assembly strategies also play a crucial role, and the appropriate choice depends on the research objective. For applications focused primarily on specific gene detection or comparative genomic analyses, Illumina-only sequencing is generally sufficient. Such studies do not aim to achieve contiguity-level completeness, and the issue of false completeness discussed in this review is therefore not applicable to these workflows. To achieve a complete genome assembly with high contiguity and accuracy, two complementary long-read–based strategies are commonly employed. The first is a hybrid approach combining ONT long reads with Illumina short reads, in which ONT provides the structural backbone for resolving long repetitive regions and Illumina reads are used to correct residual base-level errors. The second is a PacBio HiFi-based approach, in which the inherently high per-read accuracy of HiFi reads can substantially reduce the requirement for short-read polishing. ONT can produce ultra-long reads that resolve exceptionally long repetitive regions, while HiFi offers higher per-read accuracy that simplifies polishing requirements. Both strategies may encounter difficulties in recovering small plasmids, and supplementation with short-read data is generally beneficial in such cases. Consistent with the validation-centered perspective of this review, we recommend that any long-read–based assembly, including HiFi-only assemblies, undergo systematic validation and, where feasible, orthogonal short-read data may provide additional support for residual base-level correction and small plasmid recovery. The choice between these two workflows depends on platform accessibility, cost, and the length and complexity of repetitive regions to be resolved. The optimal sequencing depth depends on genome characteristics and the sequencing platform (Lerminiaux et al., 2024); however, excessively high coverage does not improve assembly quality and can reduce performance due to increased complexity and error accumulation (Rojas-Miranda et al., 2025; Wick et al., 2023). In Illumina-only sequencing, increasing coverage beyond high depths (e.g., > 100×) does not lead to further improvements in assembly outcomes. For short-read polishing, most of the benefit is achieved at approximately 25× coverage, with minimal additional gains at higher depths (Bouras et al., 2024b). Long-read sequencing has been shown to produce high-quality assemblies even at approximately 30–40× coverage using assemblers such as Flye; however, when aiming for near-complete or highly accurate genome reconstruction, substantially higher coverage, typically in the range of 100× to 200×, should be considered (Kolmogorov et al., 2019; Wick et al., 2023). In a hybrid sequencing approach, genome assembly typically involves comparative evaluation of multiple assemblers and is commonly performed by constructing a structural backbone from ONT data using assemblers such as Flye, followed by error correction with Illumina data using tools such as NextPolish.

Following assembly, a quantitative overview of assembly contiguity is obtained by comparison with a reference genome, using basic assembly statistics such as total genome size, number of contigs, and N50 values. Although N50 is widely used to summarize contig length distribution and is often interpreted as an indicator of assembly quality (Mäkinen et al., 2012), it does not directly reflect assembly accuracy. Assemblies with high N50 values can still contain misassemblies, including incorrectly merged contigs or collapsed repeat regions (Thrash et al., 2020). Therefore, assembly statistics should be regarded as technical indicators rather than definitive measures of accuracy. Subsequently, raw reads are mapped back to the assembly to assess coverage uniformity across the genome. This step enables the identification of sequence discrepancies and coverage gaps through manual inspection. Contigs exhibiting coverage substantially lower than the genome-wide average (e.g., less than half of the mean coverage) are indicative of potential contamination, whereas plasmid sequences typically display elevated coverage. In addition, graph-based visualization is employed to evaluate structural consistency and identify ambiguous regions within the assembly. Inspection of assembly graphs (e.g., GFA files) is essential to confirm that no structurally inconsistent or unsupported connections have been introduced during assembly. Finally, tools such as QUAST, CheckM, and Circlator can be employed for assembly error detection and for assessing completeness and contamination, thereby supporting the generation of a high-confidence genome (Table S1).

If structural inconsistencies are detected at any stage, reassembly is required. No universally applicable tool-based workflow currently ensures the generation of complete genomes. In cases where circularity cannot be achieved despite the use of multiple assemblers, reporting a validated draft genome is preferable to artificially increasing contiguity through parameter adjustment. Where appropriate, a complete genome may be reconstructed by ordering and orienting contigs based on alignment to a completed reference genome, although this process necessitates extensive manual curation. Genome assembly remains an inherently iterative process that requires repeated rounds of validation and refinement. As discussed above, validation approaches—including read mapping, graph inspection, reference-based comparison, and completeness assessment—should be applied in combination to evaluate both structural integrity and nucleotide-level accuracy. Although these strategies do not guarantee the generation of complete genomes in all cases, they substantially increase the likelihood of achieving high-quality assemblies. Therefore, careful evaluation and iterative improvement are critical for obtaining reliable genome sequences suitable for downstream analyses.

## Future Perspectives

Future progress will depend not only on improved sequencing and assembly algorithms, but also on the development of scalable, standardized, and transparent validation frameworks that enable researchers to assess both structural integrity and base-level accuracy. In this context, emerging computational approaches are beginning to extend the scope of assembly validation. Recent advances in machine learning and deep learning have also begun to influence genome assembly workflows, particularly in basecalling, polishing, and variant detection. These approaches offer the potential to improve assembly validation by integrating multiple sources of evidence, such as read mapping patterns, coverage profiles, and graph structures, to identify inconsistencies that may not be captured by conventional metrics. Additionally, AI-driven approaches have the potential to extend beyond base-level correction to support structural validation. For example, models trained on large-scale genome databases could, in principle, learn characteristic patterns of genome organization, including repeat structure, gene synteny, and breakpoint consistency, and identify deviations that may indicate assembly artifacts. However, distinguishing true biological structural variation from assembly artifacts remains inherently challenging, as both can produce similar signals in sequencing and assembly data. Such approaches remain largely unexplored and are likely to be limited by the availability of representative reference data and the diversity of genome architectures. Therefore, distinguishing true biological structural variation from assembly artifacts is expected to continue to require orthogonal validation strategies. As these limitations are addressed, machine learning-based validation frameworks are likely to become an important complement to existing validation strategies.

## Figures and Tables

**Table 1.** De novo assembly tools used in bacterial genome assembly

Tool	Input	Core engine	Note	Reference
ARACHNE	Short (Sanger)	OLC	Efficient scaling; primarily suited for large eukaryotic genomes	Batzoglou et al. (2002)
PCAP	Short (Sanger)	OLC (Sanger-era)	Parallel Sanger-era assembler; large-genome oriented	Huang et al. (2003)
Newbler	Short (454)	OLC	Widely used in early bacterial genome assembly	Margulies et al. (2005)
Mira	Short	OLC	Supports mapping-based assembly and polishing	Chevreux (2005)
PHRAP	Short (Sanger)	OLC (Sanger-era)	Sanger-era shotgun assembler	de la Bastide and McCombie (2007)
EULER-SR	Short (454)	DBG	A-Bruijn–inspired DBG	Chaisson and Pevzner (2008)
Velvet	Short	DBG	Early short-read DBG assembler	Zerbino and Birney (2008)
ALLPATHS-LG	Short	DBG	Optional long-read integration for gap filling	MacCallum et al. (2009)
IDBA	Short	Iterative DBG	Handles uneven coverage	Peng et al. (2010)
SOAPdenovo	Short	DBG	Scaffold-oriented; widely used for large genomes	Li et al. (2010)
SOAPdenovo2	Short	DBG	Improved memory efficiency and accuracy	Luo et al. (2012)
Minia version 3	Short	Compacted DBG	Unitig-based; evolved from Bloom filter–based approach	Salikhov et al. (2013)
MEGAHIT^*^	Short	Succinct DBG	Optimized for metagenome assembly	Li et al. (2015)
SPAdes^*^	Short	DBG	Multisized and paired-end integration	Prjibelski et al. (2020)
HybridSPAdes^*^	Hybrid	DBG	Multisized, paired-end, and long-read integration	Antipov et al. (2016b)
FALCON	Long	String graph	Diploid-aware; optimized for complex eukaryotic genomes	Chin et al. (2016)
Miniasm	Long	OLC	Consensus-free; requires polishing	Li (2016)
HINGE	Long	OLC (repeat-aware)	Improves repeat resolution using hinge-based graph construction	Kamath et al. (2017)
Canu^*^	Long	OLC	Designed for noisy long reads	Koren et al. (2017)
Flye^*^	Long	Repeat graph	Robust to complex repeats	Kolmogorov et al. (2019)
HiCanu^*^	Long (HiFi)	OLC	Improved accuracy and repeat resolution	Nurk et al. (2020)
wtdbg2^*^	Long	Fuzzy Bruijn graph	Fast and memory-efficient; designed for noisy long reads	Ruan and Li (2020)
Shasta^*^	Long (ONT)	Marker graph	Fast and memory-efficient	Shafin et al. (2020)
Raven^*^	Long	OLC	Optimized for long uncorrected reads; fast and memory-efficient	Vaser and Šikić (2021)
Hifiasm^*^	Long (HiFi)	String graph	High accuracy and repeat resolution	Cheng et al. (2021)
NECAT^*^	Long (ONT)	OLC	Efficient assembly of noisy long reads	Chen et al. (2021a)
SmartDenovo	Long	OLC	No error correction	Liu et al. (2021)
NextDenovo^*^	Long	OLC	Improved accuracy	Hu et al. (2024b)

* Tools marked with an asterisk are actively maintained and commonly used in contemporary bacterial genome assembly pipelines.

**Table 2.** Genome polishing and error correction tools

Tool	Category	Input	Core method	Key function	Reference
Pilon	Short-read polishing	Illumina reads	Read mapping	Error correction of SNPs and small indels	Walker et al. (2014)
Polypolish	Short-read polishing	Illumina reads	Multi-mapping	Repeat-aware error correction	Wick and Holt (2022)
Pypolca	Short-read polishing	Illumina reads	Read mapping	Error correction with threshold-based variant filtering	Bouras et al. (2024b)
Racon	Long-read polishing	Long reads	Read mapping	Consensus-based error correction	Vaser et al. (2017)
NeuralPolish	Long-read polishing	Long reads	Deep learning	Improved base accuracy using neural networks	Huang et al. (2021)
DeepPolisher	Long-read polishing	Long reads	Deep learning	Deep learning–based error correction	Mastoras et al. (2025)
Medaka	Long-read polishing	ONT reads	Deep learning	Signal-aware error correction	Medaka (2018)
NextPolish	Hybrid polishing	Short + long reads	Iterative polishing	Multi-platform error correction	Hu et al. (2020)

**Table 3.** Tools for genome assembly validation and quality assessment

Tool	Category	Input	Core method	Key function	Reference
REAPR	Read-based	Short reads + assembly	Read mapping	Error detection	Hunt et al. (2013)
Inspector	Read-based	Long reads	Read mapping	Structural and local error detection with correction	Chen et al. (2021b)
QUAST	Reference-based	Assembly + reference	Whole-genome alignment	Assembly quality assessment and misassembly detection	Gurevich et al. (2013)
Assemblytics	Reference-based	Assembly + reference	Whole-genome alignment	Structural variation detection	Nattestad and Schatz (2016)
CheckM	Completeness	Assembly	Lineage-specific marker genes	Completeness + contamination	Parks et al. (2015)
BUSCO	Completeness	Assembly	Marker genes (Single-copy orthologs)	Completeness assessment	Seppey et al. (2019)
KAT	k-mer based	Reads + assembly	k-mer comparison	Coverage bias, duplication detection	Mapleson et al. (2017)
Merqury	k-mer based	Reads + assembly	k-mer spectrum	Accuracy (QV) + completeness	Rhie et al. (2020)
Bandage	Graph-based	Assembly graph	Visualization	Graph inspection	Wick et al. (2015)
gfatools	Graph-based	GFA	Graph parsing	Structural analysis	Pani et al. (2024)
Circlator	Structural	Assembly	Overlap detection	Circularization validation	Hunt et al. (2015)
MOB-suite	Plasmid	Assembly	Database + typing	Plasmid reconstruction	Robertson and Nash (2018)

## Supplementary Information

Table S1. Comparative assessment of validation capabilities across genome assembly evaluation tools
