# Supplementary material for: From contiguity to accuracy: Validation-centered perspectives on bacterial genome assembly
Source: J Microbiol. 2026 Jun 19;64(6):e2604004. doi: 10.71150/jm.2604004 (PMC13577107; doi:10.71150/jm.2604004)
Supplement: Supplementary file 1 [file jm-2604004-Supplementary-Table-S1.pdf]

**Table S1.** Comparative assessment of validation capabilities across genome assembly evaluation tools

"Yes" indicates that the tool directly detects or evaluates the corresponding error type. "Partial" indicates that the tool provides indirect or limited evidence related to the error type. "No" indicates that the tool does not assess the corresponding feature.

| Tool | SNP / Small indel | Large indel | Structural misassembly | Repeat / Duplication | Completeness | Contamination | Plasmid / Circularity |
|---|---|---|---|---|---|---|---|
| REAPR | Yes | Yes | Yes | Partial | No | No | No |
| Inspector | Yes | Yes | Yes | Partial | No | No | No |
| QUAST | Yes | Yes | Yes | Yes | partial | No | No |
| Assemblytics | No | Yes | Yes | No | No | No | No |
| CheckM | No | No | No | No | Yes | Yes | No |
| BUSCO | No | No | No | No | Yes | No | No |
| KAT | No | No | No | Yes | Yes | Partial | No |
| Merqury | Yes | Yes | No | Yes | Yes | No | No |
| Bandage | No | No | Yes | Yes | No | No | Yes |
| gfatools | No | No | Yes | Yes | No | No | Yes |
| Circlator | No | No | Partial | No | No | No | Yes |
| MOB-suite | No | No | No | No | No | No | Yes |
